# Is that a belt or a snake? object attentional selection affects the early stages of visual sensory processing

**DOI:** 10.1186/1744-9081-8-6

**Published:** 2012-02-02

**Authors:** Alberto Zani, Alice M Proverbio

**Affiliations:** 1 Electro-Functional Brain Imaging unit (EFBIu), Institute of Molecular Bioimaging and Physiology, CNR, Milan, Italy; 2 Department of Psychology, University of Milano-Bicocca, Milan, Italy

**Keywords:** ERPs, C1, P1, N1, visual selective attention, visual striate cortex, V1, space and object parallel processing, shape processing time course, shape categorization

## Abstract

**Background:**

There is at present crescent empirical evidence deriving from different lines of ERPs research that, unlike previously observed, the earliest sensory visual response, known as C1 component or P/N80, generated within the striate cortex, might be modulated by selective attention to visual stimulus features. Up to now, evidence of this modulation has been related to space location, and simple features such as spatial frequency, luminance, and texture. Additionally, neurophysiological conditions, such as emotion, vigilance, the reflexive or voluntary nature of input attentional selection, and workload have also been related to C1 modulations, although at least the workload status has received controversial indications. No information is instead available, at present, for objects attentional selection.

**Methods:**

In this study object- and space-based attention mechanisms were conjointly investigated by presenting complex, familiar shapes of artefacts and animals, intermixed with distracters, in different tasks requiring the selection of a relevant target-category within a relevant spatial location, while ignoring the other shape categories within this location, and, overall, all the categories at an irrelevant location. EEG was recorded from 30 scalp electrode sites in 21 right-handed participants.

**Results and Conclusions:**

ERP findings showed that visual processing was modulated by both shape- and location-relevance *per se*, beginning separately at the latency of the early phase of a precocious negativity (60-80 ms) at mesial scalp sites consistent with the C1 component, and a positivity at more lateral sites. The data also showed that the attentional modulation progressed conjointly at the latency of the subsequent P1 (100-120 ms) and N1 (120-180 ms), as well as later-latency components. These findings support the views that (1) V1 may be precociously modulated by direct top-down influences, and participates to object, besides simple features, attentional selection; (2) object spatial and non-spatial features selection might begin with an early, parallel detection of a target object in the visual field, followed by the progressive focusing of spatial attention onto the location of an actual target for its identification, somehow in line with neural mechanisms reported in the literature as "object-based space selection", or with those proposed for visual search.

## Introduction

In the literature on neural mechanisms underlying visual selective attention, the theoretical view that attention is indeed unable to modulate V1 activity is so generally acknowledged to be reported in available cognitive neuroscience handbooks. Several influential studies have contributed to this view. For example, Martinez et al. [1] reported no attentive modulation for the early C1 component, originating in the striate cortex, in a combined ERPs and fMRI spatial attention study, but a facilitation of attended signals at P75 level within the extrastriate visual cortex. The lack of an attentional modulation of ERPs C1 led the authors to hypothesize that the spatially-based modulation of the striate cortex hemodynamic activity reported in the same study might represent a delayed, re-entrant feedback from higher-order visual areas or a sustained biasing of striate cortical neurons by the fronto-parietal network. In line with these findings, other electrophysiological studies on visual selective spatial attention did not show any attentional effects before 90 ms in the amplitude of C1 component [2,3]. In a review of spatial and temporal properties of neural activity during visual selective attention [4] it was proposed that paying attention to spatial location enhanced the activation of the extrastriate occipital areas (V2, V3/VP), as reflected by a greater amplitude of P1 (80-130 ms) and N1 (140-200 ms) components, generated in those areas. On the other hand, the selection of non-spatial features, such as color, shape, spatial frequency, orientation, direction of motion, was not associated with a modulation of the earlier sensory responses, but only with later negative ERP deflection, called "selection negativity" (SN) or N2p responses (150-250 ms). Consistently, single cell recording studies in monkeys had shown robust attentional effects in the extrastriate occipital cortex, but not in the V1 striate cortex [e. g., [5,6]]. In these studies, however, stimulus material was much larger than V1 receptive fields, thus possibly explaining the lack of attention effects in the primary visual cortex.

In those same years, however, evidence from different methodological lines of research in cognitive neuroscience had appeared hinting at the hypothesis that, unlike generally believed, visual attention might instead have been somehow able to modulate the processing of visual input already starting in the primary visual cortex [7,8]. For instance, spatial attention effects had been reported in the striate occipital cortices as indexed by single-unit recordings [e. g., [9,10]]. Interestingly, in more recent years further single-unit studies have also indicated direct influences of attention on the processing of visual inputs already beginning at the primary visual cortex (V1), if not the lowest neuroanatomical levels of the visual system. A recent single-unit recording study by McAdams and Reid [11] in macaques found, for instance, that attention was able to enhance the visual responses of simple cells in the primary visual cortex (V1) in between 23.5-47 ms post-stimulus, without changing the underlying spatial and temporal structure of the receptive field. Still more recently, McAlonan, Cavanaugh, and Wurtz [12] have published an authorative study stating that spotlight attention modulated the processing of visual signals before they even reached the visual cortex by increasing neuronal responses in the thalamic lateral geniculate nucleus, and by decreasing responses in the adjacent thalamic reticular nucleus, as earliest as in between 20-40 ms post-stimulus.

Neuroimaging had also contributed to this matter: indeed, functional magnetic resonance imaging (fMRI) studies had shown a modulation of the primary visual cortex during spatial attentional processing [13]. Such a modulation would occur in the absence of visual stimulation too [14,15]. Additionally, a meta-analysis of PET studies on selective attention to non-spatial features (such as colour and size) reported a neural modulation of several occipito-temporal areas including BA17 [16]. Other fMRI research contributed to explain separate mechanisms of attentional suppression for unattended inputs and of attentional facilitations for attended ones in striate and extrastriate cortex [17].

Nevertheless, because of the inadequate temporal resolution of fMRI technique, it had not been possible to understand whether the observed modulation of V1 activity occurred at the earliest latency level or was the result of a delayed re-entrant feedback from the higher-order occipital-temporal and frontal-parietal regions.

At this regard, electrophysiological studies revealed to be quite helpful in providing the time course of brain activation [2,18]. However, based on controversial findings in the literature about the timing and the anatomical level of the visual system at which attention affects visual processing the debate on this matter is still opened. Indeed, an ERP study by Fu et al. [19] investigating the interaction of voluntary allocation of attention and perceptual load on the modulation of visual processing found significant effects for the N1 (190 ms) but not for the P1 (100 ms) or the earlier C1 (84 ms) component. Conversely, the combination of an involuntary allocation of visual attention and perceptual load positively contributed to the earliest C1 attentional modulation, and the involvement of visual striate cortex in two following studies by the same experimental group [20,21], so that different neural mechanisms of modulation of early sensory visual processing for voluntary and involuntary spatial attention have been advanced for giving reason of these apparently idiosyncratic findings. Unlike these studies, an ERPs study by Kelly et al. [22] provided evidence of a robust enhancement at C1 level elicited by voluntary spatial attention, using a cuing task in which standard and target stimuli were presented either at relevant or irrelevant locations across the upper and lower visual hemifields. These attention effects, which source analyses attributed to a generator in the striate cortex, started around 50-60 ms from the stimulus onset, and were not affected by the visual fields, neither *per se*, or in interaction. Furthermore, evidence of a lower C1 negativity (i. e., greater positivity) under high attentional load, with respect to low-load, for distracters in the upper, but not in the lower, visual hemifield during a voluntary attention task has also been reported [23]. It is interesting that, overall, these findings seem to hint at possible attention mode and/or stimulus-related differences in the C1 modulation.

As for the object-based attention, in the pioneering study by Zani and Proverbio [24], ERPs to attentionally relevant and irrelevant check sizes were compared; it was found an enhanced lateral-occipital P90 along with a mesial-occipital N115 negativity to relevant spatial frequencies. Attention modulation of the P1 response was later on reported for the conjoined selection of location and spatial frequency features [25]. Moreover, Proverbio et al. [26] investigated stimulus orientation selection and found attention effects at P1 (80-140 ms) post-stimulus latency. Later on, a C1 modulation by visual attention has been also reported for the selection of competing stimulus attributes. For instance, in a study involving the selection of one of two transparent superimposed surfaces, Khoe and colleagues [27] found a modulation of a negative C1 (75-110 ms) and N1 (160-210 ms) components for the relevant vs. the irrelevant translational surface within an attended space location. Most interestingly, an influential study by Karns and Knight [28] also reported a modulation of the early phase of C1 (62-72 ms), besides of the subsequent P1 and N1, in an intermodal spatial selection task in which the stimuli of the sensory modality to be attended were all presented within the same attended location.

Further studies investigating visual spatial and non-spatial features-conjunction, voluntary selection presenting relevant and irrelevant spatial-frequency gratings at relevant and irrelevant quadrants of the visual field to sizeable samples of subjects, so to obtain an high signal-to-noise ratio, also indicated robust modulating effects on the earlier C1 component, besides the subsequent P1, analysing the C1 and P1 amplitudes either as mean area values centred over their peak latencies [29] or in separate 20 ms time spans in between 60-140 ms post-stimulus [30]. Interestingly, these ERP data are in line with previous findings obtained on a small group of participants, and reported in a published meeting abstract [31]. Most importantly, a rather recent source reconstruction study based on a high-resolution electrode montage (i.e., 128 channels) proved that the modulation of the C1 response, found, independent of the visual field, at the earliest post-stimulus latency (40-60 ms) during the aforementioned voluntary visual feature-conjunction selection tasks, arose, beyond other areas, from the BA17 sub-region of the cuneus, in the visual primary areas [32].

Most interestingly, scant findings have also been described of an ERP P1 smaller amplitude to feature-relevant than to feature-irrelevant stimuli presented at a neglected location [33,29], possibly lending an indicative timing to the aforementioned fMRI-indexed mechanisms of attentional suppression and facilitation for unattended and attended input, respectively [17].

Given the renown anatomical variability of striate cortex (C1 generator), we investigated whether inter-individual differences in VEP morphology might affect the nature and the polarity of C1 response and its attentional modulation [34]. While attention effects resulted in an increased positivity at both C1 and P1 level in a sub-group of subjects that exhibited a prominent P80, shape relevance was associated with an enhanced negativity at C1 level and a smaller P1 component in the sub-group that exhibited a prominent N80. Notwithstanding the difference in the polarity of sensory response (either P80 or N80), it was therefore found that spatial attention increased the positivity of evoked potential, whereas feature-based attention increased the negativity of N80 response.

It is interesting to consider that the C1 modulation by visual attention has been also reported for the selection of the affective or the linguistic content of visual stimuli. As for the effect of stimulus emotional value, Stolarova et al. [35] found a difference in the modulation of C1 elicited by aversive vs. neutral stimuli at 65-90 ms post-stimulus, suggesting an involvement of primary visual areas in affective evaluations. This finding agrees with others in the literature supporting the notion that C1 response (generated in the striate cortex) is modulated by the affective valence of stimulus [36]. Object content, matched for perceptual characteristics such as size, luminance, and spatial frequency distribution, is also known to affect the earliest sensory stage. For example, Proverbio et al. [37] obtained larger P1 to faces than equiluminant familiar objects. Similarly it was found a larger P1 to complex IAPS scenes displaying humans rather than unanimated landscapes [38]. In addition, Proverbio and Adorni [39] found larger C1 (i. e., 70-90 ms) to words during an orthographic (letter detection) vs. a lexical decision (word/pseudoword discrimination) task.

In summary, there are at present several studies in the neuroscientific literature reporting an early timing of attention modulation of C1 and P1 responses during spatial selection, attention to spatial frequency [18,29,32], luminance, texture, emotion, workload, although still unknown whether in interaction with either voluntary or involuntary allocation of visuospatial attention [19-21,23,40], stimulation context [41], task [39], and vigilance. Much fewer studies investigated stimulus features selection *per se*, and still fewer object selection. Among the few studies investigating shape selection, Taylor [42] used simple geometric forms of different colors as target stimuli finding that object-based attention can affect the latency and amplitude of the P1 component, as a function of task requirements. Purpose of the present study was to investigate how early attention affects object processing, or, more specifically, the earliest time and neuroanatomical level at which, in the progression of hierarchical levels in the visual system, attention might boost object processing modulating on-line neural activity. To this goal, we adopted conjoined space and shape selection tasks in which participants had to pay selectively heed to one target-category of familiar shapes sequentially presented intermixed with shapes from two other increasingly conflicting categories, balanced for luminance and perceptual familiarity, presented at a relevant spatial location, while at the same time totally ignoring these same categories as presented at an irrelevant location. We hypothesized that, had the visual striate cortex level of processing, besides the extrastriate level, be directly interested in the attentional selection of these complex shapes (despite their small receptive fields and via details analysis), this would have been manifested at the scalp in a modulation of the earliest post-stimulus visual C1 response.

Being our goal the investigation of the timing and neural level at which visual attention might start affecting object processing, and not the timing of object categorization *per se*, to increase signal relative to noise so to reliably measure the earliest C1, besides the later, P1 activations, for each volunteer ERPs were averaged as a function of the conjoined object and space relevance conditions independent of the target-category (animals vs. artefacts) and of the visual hemifield (left and right). Empirical evidence in the ERPs literature pertaining to object processing and categorization supports this choice. Indeed, a pioneering study indicated that a divergence in ERP waveforms to target- vs. non-target differential images, observed at frontal electrode sites only, did not develop earlier than about 150 ms after image onset [43]. Later on, ERP signs of object categorization processes at posterior occipital-temporal electrode sites have also been demonstrated, but not before the N1 latency range (e. g., 130-180 ms) after stimulus-onset [44,45]. This procedure also stands on the acknowledged tenets that the deployment of attention follows similar neural mechanisms across the vertical and/or horizontal meridians [see e. g., [22,25,29,30,46,47]], or that the effects of stimulus features (e. g., spatial frequency or colour) do not interact with the attentional relevance of the latter [25,30,32,46]. Last but not least, for pursuing a good signal-to-noise ratio a sizeable sample of participants was tested.

## Methods

### Participants

Twenty one (11 males, 10 females) unpaid university students took part in the present study, approved by the Italian National Research Council (CNR) Ethical Board, and conducted in accordance with APA ethical standards for the treatment of human experimental volunteers (American Psychological Association, 1992). All signed a written informed consent for participating in the study in compliance with the indications of Declaration of Helsinki (BMJ 1991; 302: 1194). Unfortunately, three of them (i. e., two (2) males and one (1) female) had to be subsequently discarded from ERP analyses for excessive muscular and ocular artefacts. Hence, a sizeable sample of 18 volunteers remained. The mean age of the 18 participants was about 22.5 years. All were right-handed and had normal or corrected-to-normal vision, as well as normal hearing.

### Stimuli

**Figure 1:**
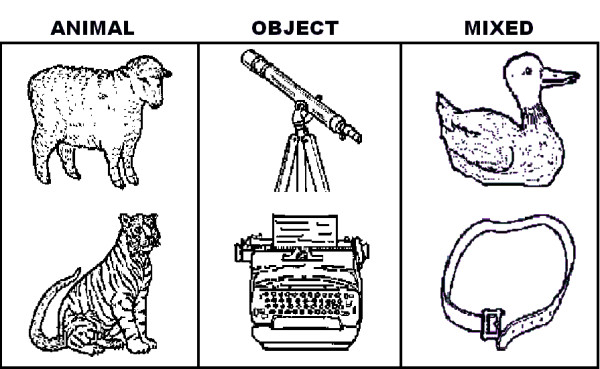
**Example of stimuli belonging to the animal, artefact or distracter categories**.

Stimulus set comprised 672 pairs of stimuli, presented vertically arrayed on the right or left visual hemifields of a remote display monitor of a PC used for volunteers' stimulation. Stimulus pairs were made up of B/W drawings representing in a schematic but realistic manner 44 different animals and 44 different familiar artefacts randomly combined across them (see Figure 1 for some examples). In this way, 168 pairs of animals, 168 homogeneous pairs of artefacts as well as 336 pairs of mixed stimuli (animals and artefacts) were built up. Animals, artefacts and mixed pairs had the same average luminance, as shown by an ANOVA performed on luminance values obtained by means of a Minolta CS-100 photometer (p < 0.145; animals = 18.28, objects = 17.81 and mixed pairs = 17.8 candles/m^2^). Half of the mixed-pairs showed an animal in the upper visual field and an artefact in the lower visual field, and vice versa for the remaining pairs. Stimulus pairs size were 6°22' 12'' in height × 3°49' 12'' in width. They were flashed in the left or right visual hemifields beginning at 2°30' of eccentricity from the vertical meridian, centered on the horizontal meridian. Stimulus duration was 250 ms with an ISI ranging between 900-1200 ms.

With these stimulus pairs eight (8) blocks of stimulus trials were built up, each consisting of 84 trials of stimuli and lasting 2 minutes. Each block of 84 trials was subdivided in an equal number of 28 animal, artefact, and mixed category-pairs, half of which equally fell in the right and left space hemifields. Trials order changed randomly from block to block. In each sequence three warning signals (i.e., SET, READY, GO) additionally preceded the true stimulus trials, inviting the participants to concentrate and get ready to perform the visual attention selection task.

### Procedure

Volunteers sat on a comfortable armchair placed in an electrically and acoustically shielded, dimly lit, cubicle at the viewing distance of 140 cm from the stimulation PC display monitor. They were instructed to fixate a cross at the center of the display and avoid any eye or body movements. On each block of trials, the shape-pairs belonging to the three (3) different categories were sequentially presented one at the time in random order either within the left or right hemifields of the stimulation PC display monitor. Different conjoined selective attention conditions were administered in randomized order for either the animal or artefact category-pairs and the space hemifields.

We chose to use the attentional selection of "living" vs. "not living" stimulus materials because the latter paradigm is much used in image processing and object categorization studies [see [48]]. We added a mixed pair category because we wanted that the participants deployed their attentional resources onto the target category at the relevant location through an effortful discrimination of increasingly conflicting features across shape-pairs categories, i. e., relevant (S+), distracters (S+/-), irrelevant (S-). Indeed, this addition introduced a higher, or relatively higher, perceptual processing load than in previous studies, as in some recent studies investigating the influence of perceptual load on C1 modulation in relation to voluntary and/or involuntary allocation of visuospatial attention [19-21,23]. This also made our space-based object selection task somehow consistent with the simple-features conjoined selection task we used in previous studies, where participants had to discriminate between spatial frequency gratings in the same bandwidth (e. g., 0.75 and 1.5 c/deg, or 3 and 6 c/deg) presented across the relevant and irrelevant quadrants of the visual fields to perform their conjoined spatial and non-spatial features selection [25,29,30,32].

In half of the blocks of trials the volunteers were instructed before the starting of the attention-run to pay selectively heed and motorically respond to the animal-images category within the relevant spatial hemifield (i. e., left or right), while ignoring the other two image-category-pairs within that hemifield, and, overall, all the stimulus conditions at the opposite, irrelevant hemifield. In the other half of blocks the artefact-category-pairs had to be attended and responded at the left or right hemifields. Therefore, although the physical stimuli remained unchanged, from run to run attention shifted conjointly across spatial location and image category-pairs.

This way, independent of the image-category and visual hemifield, and according to the attention condition, the shape-category-pairs could be: relevant (L+) or not (L-) in spatial location, and relevant (S+), in between relevant and irrelevant (S+/-) or irrelevant (S-) in shape category. More specifically, in separate conjoined-attention relevance combinations the same images pair could be relevant both in location and shape category (L+S+); relevant in space but completely irrelevant in shape (L+S-); relevant in space but half-relevant/half-irrelevant (distracter) in shape (L+S+/-); irrelevant in space and relevant in shape (L-S+); irrelevant in space and completely irrelevant in shape (L-S-); irrelevant in space and half-relevant/half-irrelevant (distracter) in shape (L-S+/-). Participants were trained to respond as accurately and quickly as possible by pressing a button to targets with either the left or right index finger. Task conditions and responding hands were randomized and balanced across and within subjects.

### EEG acquisition and analysis

Electroencephalogram was recorded from 30 scalp sites by means of Ag/AgCl electrodes mounted in an elastic cap. The electrodes were located at pre-frontal (Fp1, AFz, Fp2), frontal (F7, F3, Fz, F4, F8), fronto-central (FC1, FC2, FC5, FC6), central (C3, Cz, C4), central parietal (CP5, CP6), temporal (T3, T4), posterior temporal (T5, T6), parietal (P3, Pz, P4), occipito-parietal (PO3, PO4), mesial occipital (O1, O2), lateral occipital (PO7/OL, PO8/OR) scalp sites of the International 10-20 System. To ensure that eye-fixation was maintained, horizontal and vertical oculograms (EOG) were also recorded by two electrodes placed below and above the right eye (VEOG) and two electrodes placed at the outer canthi of the eyes (HEOG). Discrete single trials-related EEG sweeps were recorded on-line starting from 50 ms before to 800 ms after stimulus presentation. EEG epochs were synchronized with the onset of stimulus presentation and they were digitized at a rate of 512 samples per second. Amplifier filters were set-up at 0.16-50 Hz for EEG channels, and 0.02-50 HZ for EOG channels. Electrode impedance was kept below 5 kΩ. The reference lead was linked earlobes, whereas a pre-frontal electrode served as ground site.

EEG sweeps for each block of trials were stored to separate digital files in the HD of a "master" PC. These files were automatically averaged offline using an artifact-rejection procedure to discard epochs in which eye movements, blinking, excessive muscle potentials or amplifier saturation occurred. The criterion for artifact rejection was peak-to-peak amplitude exceeding +/-70 μV for EEG signal and +/-100 μV for EOG signal, and the rejection rate across subjects was overall about 5%. EEG sweeps related to incorrect behavioral responses (i. e., false alarms, FAs), mostly concerning the "distracters" attention condition at the relevant location (L+S+-; see the "Behavioral results" Section below for further information), were also discarded from averaged ERPs.

Since, how indicated in the "Introduction" section, we sought to investigate the timing and neural level at which visual attention affects object processing, and not the timing of object categorization *per se*, for each volunteer ERPs were obtained as a function of the six (6) conjoined attention-relevance conditions collapsing data relative to the two target-image-categories (animals vs. artefacts) and the two visual hemifields (left and right).

Then, for each volunteer, an ERP average waveform for the half-relevant shape-category-pairs at the relevant space location (i. e., L+S+/-) was obtained including, on average, about 103-106 trials, while average ERPs for the remaining attention conditions included, instead, about 107-110 trials. In addition, grand-average ERP waveforms for "location-relevance" (i.e., L+ and L-) and "shape-relevance" conditions (i. e., S+, S-, and S+/-) *per se *were computed, the former including in between 317-326 trials for the relevant location and about 321-330 trials for the irrelevant one, respectively, due to the collapsing of the three (3) shape-relevance options, and the latter made up of about 214-220, 206-212, and 214-220 trials, respectively, due to the collapsing of the two location-relevance alternatives. Importantly, due to the grand-averaging across the sample of 18 volunteers, grand-average ERPs made up of thousands of trials were also obtained and plotted in the figures for illustrating our findings.

To further improve the signal-to-noise ratio, and to ensure that any possible "noise" in the ERP signals preceding stimulus delivery across experimental conditions could not affect the post-stimulus measures obtained, average ERPs were submitted to frequency-based band-pass digital filtering using low- and high-pass settings of 0.16 and 30 Hz, respectively, and with a roll off of 12 dB/octave. While cleaning up ERP waveforms without producing any phase shifts, which are a characteristic of electronic filters, this band-pass filtering let freely pass only the frequencies lower than 30 Hz, that is, those frequencies, and especially alpha (8 - 15 Hz) and beta (15 - 30 Hz) bands, which have been identified as mechanisms by which selective attention is deployed within vision [49-53].

Early ERP components of interest were measured as the mean amplitude in a given time window. In detail, the mean amplitude of C1 and P1 responses was measured at mesial-occipital (O1, O2), lateral-occipital (OL, OR) posterior temporal (T5, T6) and occipito-parietal (PO1, PO2) sites in three (3) different, short time windows (60-80, 80-100, 100-120 ms). Conversely, mean amplitude values for N1 component were computed at mesial-occipital (O1, O2), lateral-occipital (OL, OR), occipito-parietal (PO1, PO2) and posterior temporal (T5, T6) sites, centered on the peak amplitude latency range of 140-160 ms. Finally, the P3/N400 and LP (*late positivity*) components mean amplitude was measured at parietal sites (P3, Pz, P4), where they reached their maximum values, in the 380-450 ms and 450-520 ms latency windows, respectively.

The aforementioned amplitude measures were subjected to multifactorial repeated-measures ANOVAs. Factors included: (1) location relevance: L+ (attended location), L- (unattended location); (2) shape relevance: S+ (target pairs), S- (non-target pairs), S+/- (mixed pairs); (3) electrode site: as a function of ERP component of interest; (4) hemisphere (right and left). Post-hoc comparisons among means were performed by means of Tukey or Newman-Keuls tests. The Greehouse-Geisser correction was applied to compensate for possible violations of the sphericity assumption associated with factors which had more than two levels. In this case, the degrees of freedom accordingly modified are reported together with the epsilon (ε) and the corrected probability level.

Topographical scalp current density (SCD - i. e., second spatial derivative of the potential) maps were computed from the spherical spline-interpolation of the surface voltage recordings between scalp electrodes at specific latencies. These SCD maps were plotted as saturation-level-coded values of a three red-black-green colours-scale.

## Results

### Behavioural Results

A three-way repeated-measures ANOVA with target-category-pairs (animals Vs artefacts), spatial hemifield (left or right), and response hand (left or right) as main factors was carried out on average reaction times (RTs). This analysis indicated that the volunteers were overall significantly [F(1, 17) = 61.669, p < 0.001; ε = 0.98] much quicker to respond to animals (M = 517 ms; SD = 16) than artefacts (M = 564 ms; SD = 18), and that neither the visual hemifield or the response hand factors affected motor response speed *per se *or in interaction.

A further three-way repeated measures ANOVA on the arc-sin-transformed percentage of false alarms (FAs) was carried out with spatial location (relevant vs. irrelevant), target-category (animal vs. artefact), and shape-pairs (animal, artefact, mixed) as main factors. Because of the small or null amount of FAs obtained across conditions, spatial-location factor levels (i. e., relevant vs. irrelevant) for these behavioural mistakes included data collapsed across the left and right visual-hemifields. The ANOVA yielded a highly significant interaction among the three factors [F(2, 34) = 1125.17, p < 0.0001; ε = 1]. This interaction proved that FAs percentage was significantly increased by location relevance, it being overall higher for the relevant than irrelevant location. In addition, at the relevant location only, when artefact-image-pairs were the target-category volunteers' percentage of mistaken motor responses to mixed-image-pairs was higher (M = 5.69%) than when animals-image-pairs were the target-category (M = 1.78%). However, no differences were instead found within the relevant location between the almost negligible number of FAs obtained for the animal- and artefact-image pairs (0.3% vs. 0.7%) as a function of the opposite target-category selection.

Overall, these behavioural findings strongly suggest that, as expected, at this late processing level the volunteers conjointly deployed visual attention processing resources onto the salient target-category within the relevant spatial location mostly neglecting visual input from the irrelevant location. In our view, they also suggest that the volunteers focused their attentional resources almost exclusively on the target-shape category, hardly dispersing them onto the mixed-pair distracters, and still less on the shape-irrelevant category. However, both these behavioural measures are not pure signs of the visual selective processing because they include also signs of the processing stages required for response selection and execution. Hence, they do not directly index the timing and the mechanisms of allocation of early visual attentional processing well preceding the motor output, this timing being provided instead, if anything, by ERPs only.

### Electrophysiological Results

In Table 1 a summary is reported of the significances of attentional effects relative to shape and location relevance, or their interaction, attained in the ANOVAs carried out on the mean amplitudes of the separate time windows for the earliest processing levels. Overall, these significances robustly suggest an attentional modulation for both shape-based (especially at mesial-occipital electrode sites (O1 and O2) and location-based (at more lateral sites) visual-selection starting at the earliest post-stimulus latency, and, possibly, anatomical visual processing levels. Although having different functional meanings and neuroanatomical substrates, these conjoined attentional modulations were also found for the later latency ERP components, as summarized in Table 2. Below, a detailed report of these findings is provided as a function of the progressing processing time.

**Table 1:** Attentional effects for shape and location relevance (in the occipital-temporal cortex) for the three early latency processing windows considered.

Time Window	C1 (60-80 ms)	C1 (80-100 ms)	P1 (100-120 ms)
**Significant Factors**			
Location Relevance	p < 0.024	p < 0.01	p < 0.003
Shape Relevance × Electrode	p < 0.027	n.s.	n.s.
Location × Shape Relevance	n.s.	p < 0.01	n.s.
Location × Shape Relevance × Electrode	n.s.	n.s.	p < 0.028
Shape Relevance × Hemisphere	n.s.	n.s.	p < 0.05
Location × Shape Relevance × Hemisphere	n.s.	n.s.	p < 0.05

**Table 2:** Attentional effects of shape and location relevance (in the occipital-temporal cortex) for the later-latency time windows investigated.

Time Window	N1 (140-160 ms)	P300/N400 (380-450 ms)	Late positivity (450-520 ms)
**Significant Factors**			
Location Relevance	p < 0.030	p < 0.001	p < 0.0000
Shape Relevance	n.s.	p < 0.001	p < 0.001
Shape Relevance × Electrode	n.s.	p < 0.001	n.s.
Location × Shape Relevance	n.s.	p < 0.002	p < 0.0004
Shape Relevance × Hemisphere × Electrode	p < 0.048	n.s.	n.s.

#### C1 (60-80 ms)

**Figure 2:**
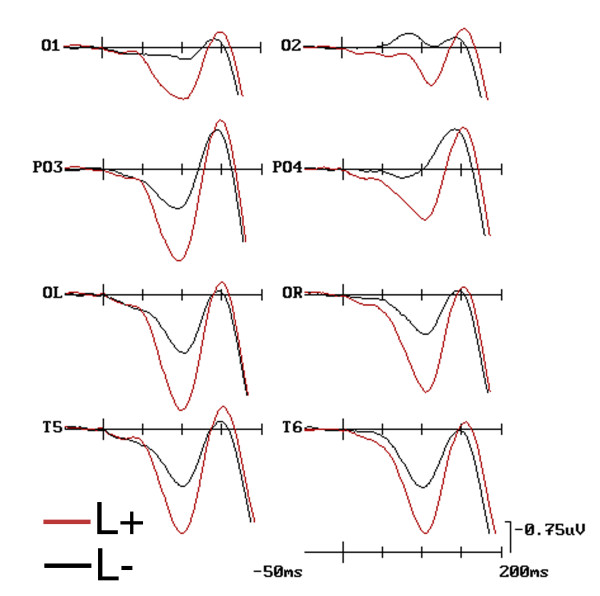
**Grand-mean ERPs as obtained at left and right mesial-occipital, occipital-parietal, lateral-occipital, and temporal electrode-sites as a function of shapes location relevance (i. e., L+ and L-) collapsing data across participants, target-categories (i. e., animals and artefacts), and shape relevance conditions (i. e., S+, S+/-, and S-)**. Note that here only the early-latency ERP responses have been plotted with an expanded time scale of 200 ms and tick-mark progressions of 50 ms to bring out the earliness of the modulation of sensory-related C1, P1, and N1 components by spatial selective attention.

**Figure 3:**
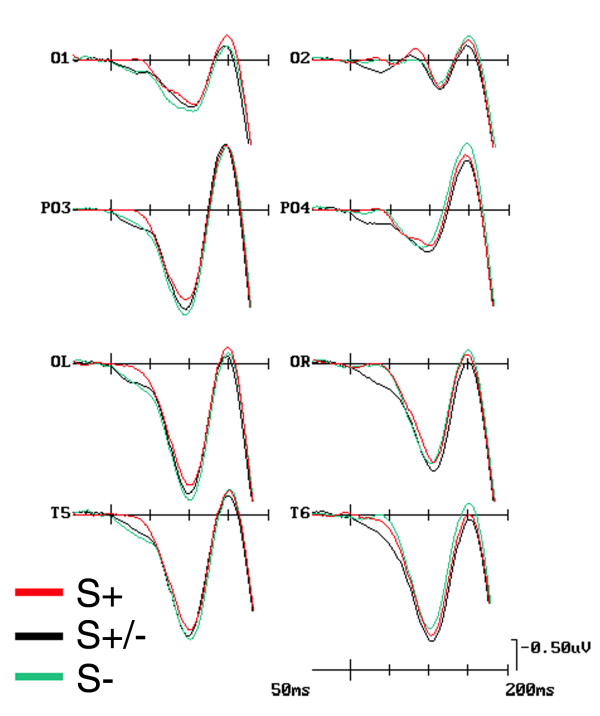
**Grand-average ERPs at left and right mesial-occipital, occipital-parietal, lateral-occipital, and temporal electrode-sites as a function of shape relevance conditions (i. e., S+, S+/-, and S-) *per se***. These ERPs were obtained averaging data across participants, target-categories (i. e., animals and artefacts), and shapes location relevance (i. e., L+ and L-). Scaling is the same as for Fig. 2.

**Figure 4:**
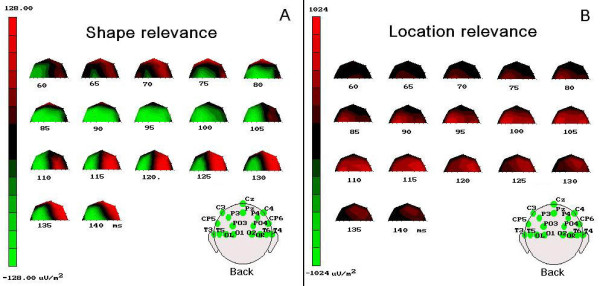
**(A) Time series (pass = 5 ms) of topographical maps (back view) plotting the 3-red-black-green-colours saturation-coded surface scalp current density (SCD) values computed on the difference waveform obtained by subtracting ERPs to shape-irrelevant pairs (S-) from ERPs to shape-relevant pairs (S+)**, **(**B**)Same time series maps as for A, except that SCD values were computed on the difference voltage obtained by subtracting ERPs to location-irrelevant pairs (L-) from ERPs to location-relevant pairs (L+).** It is worthy of note that, overall, shape-relevance manifested as a negative SCD mostly concerning the mesial-occipital electrode sites, whereas location-relevance determined a strong positive SCD mostly concerning the lateral occipital, posterior temporal, and parietal electrode sites.

C1 was of greater amplitude (more positive) to pairs presented in the relevant than in the irrelevant location ['Location': F(1, 17) = 6.171, p < 0.024; L+ = 1.25 μV, SD = 0.39 vs. L- = 0.48 μV, SD = 0.37], as visible in Figure 2. The ANOVA also yielded significant effects for 'Shape' relevance although in interaction with the 'Electrode' factor [F(4, 77) = 3.21, p < 0.028; ε = 0.718], indicating that, independent of location relevance, shape-pairs relevance (S+ = 0.22; SD = 0.35) produced a more negative C1 than shape-irrelevance (S- = 0.46; SD = 0.39; p < 0.01), but not distracters, at mesial-occipital sites only, as can be seen from the ERP waveforms related to these conditions, plotted in Figure 3. That these object-selection effects already arose, in parallel with location relevance, at this earliest timing with this well-defined mesial scalp topographic distribution is also strongly advocated by the time-series topographical mapping of Figure 4A. Conversely, there was no difference between C1 to targets and distracters (S+/- = 0.21; SD = 0.37).

C1 was more positive over the right than left hemisphere ('Hemisphere': F(1, 17) = 6.02, p < 0.039; LH = 1.09 μV, SD = 0.29; RH = 0.54 μV, SD = 0.33). Moreover it was more negative at mesial-occipital electrode sites (O1/2 = 0.30 μV, SD = 0.35) than at all other sites (OL/R = 1.17 μV, SD = 0.36; T5/6 = 1.10 μV, SD = 0.22; PO3/4 = 0.97 μV, SD = 0.29), as proved by the post-hoc comparisons carried out for the significant 'electrode' factor significance (F(3, 33) = 11.021, p < 0.000642; ε = 0.652). These effects can once again be clearly appreciated by looking at the maps of Figure 4A.

#### C1 (80-100 ms)

**Figure 5:**
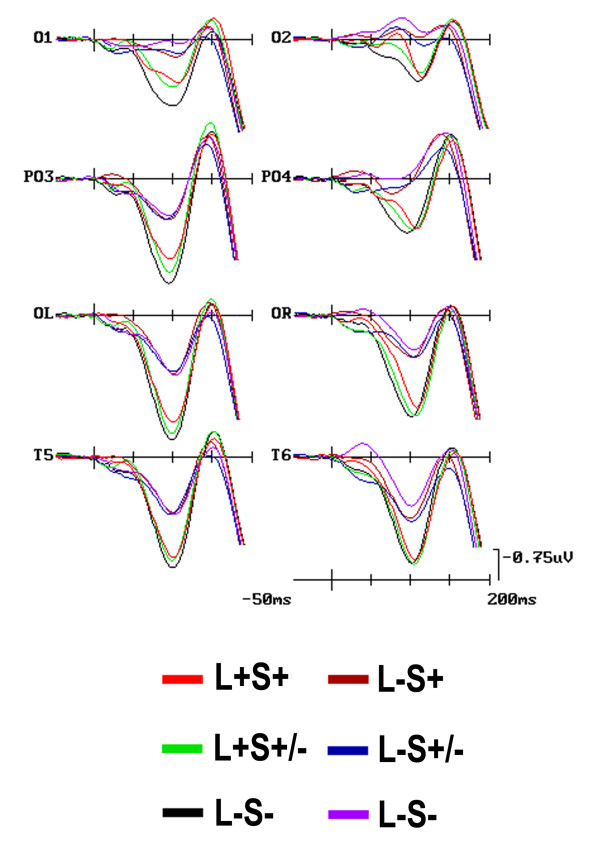
**ERP waveforms at left and right mesial-occipital, occipital-parietal, lateral-occipital, and occipital-temporal electrodes grand-averaged across participants as a function of the conjoined spatial and non-spatial relevance of shape-pairs (i.e., L+S-, L+S+/-, and L+S-, as well as L-S+, L-S+/-, and L-S-)**. Whereas C1 was of greater amplitude over mesial occipital sites, P1 response was larger over lateral occipital sites. Scaling is the same as for Figures 2 and 3.

C1 amplitude was clearly affected by 'Location relevance' *per se *(F(1, 17) = 9.01; p < 0.01) at this time window too, in that shapes falling at the relevant location (L+ = 2.41 μV; SD = 0.67) elicited a greater positivity than those falling at the irrelevant one (L- = 0.91 μV; SD = 0.41), as visible in maps of Figure 4B. 'Shape relevance' was also significant, but only in interaction with 'location relevance' [F(2, 29) = 6.01, p < 0.01; ε = 0.85]. These conjoined effects of visual attention are made evident by the ERP waveforms plotted in Figure 5. As confirmed by post-hoc comparisons, this interaction indicated that at this time, unlike previously, the visual system deployed an increased neural processing onto the relevant shapes (S+ = 1.95 μV; SD = 0.83) than onto both the irrelevant shapes (S- = 2.98 μV; SD = 0.79; p < 0.001) and distracters (S+/- = 2.67 μV; SD = 0.77; p < 0.001), narrowing this differential deployment to the relevant location only (L+).

The significance of 'Electrode' [F(2, 34) = 20.27; p < 0.000001, ε = 0.67] factor and the interaction of 'Electrode × Hemisphere' [F(2, 36) = 3.508, p < 0.022; ε = 0.70] also showed how C1 was overall more positive (P80) at posterior-temporal (2.55 μV; SD = 0.56), lateral-occipital (2.41 μV; SD = 0.66), and parietal-occipital sites (1.68 μV: SD = 0.62), especially of the right hemisphere, and most negative (N80) at the mesial occipital sites (0.39 μV; SD = 0.62) as can be seen looking at Figure 4B again.

#### P1 (100-120 ms)

At this later processing stage, image pairs falling at the relevant location yielded a greater P1 than those falling at the irrelevant one [F(1, 17) = 11.1, p < 0.003; L+ = 2.29 μV, SD = 0.59 vs. L- = 1.01 μV, SD = 0.47]. This component was also sensitive to the interaction of 'Location × Shape × Electrode' (F(5, 91) = 2.65, p < 0.028; ε = 0.89). Post-hoc comparisons made evident that, despite a topographically distributed general trend at all posterior sites, the lower positivity shown by relevant-shapes in comparison to irrelevant-ones at the relevant location reached significance at the mesial-occipital (L+S+ = 1.01 μV; SD = 0.70; L+S- = 1.61 μV; SD = 0.63) and lateral-occipital (L+S+ = 2.28 μV; SD = 0.70; L+S- = 2.66 μV; SD = 0.63) electrode sites only, as clearly visible in grand-mean ERPs averaged across subjects plotted in Figure 5.

A further triple interaction between 'Location', 'Shape' and 'Hemisphere' factors also reached significance [F(2, 26) = 3.37, p < 0.05; ε = 0.77]. This interaction revealed that the smaller mean positivity recorded in response to the shape-relevant condition (S+ = 2.06 μV, SD = 0.62) than the shape-irrelevant one (S+ = 2.53 μV, SD = 0.59) for the relevant location was significant, independent of electrode sites, at the left-hemisphere only (see Figure 5).

However, and most interestingly, the effects on P1 mean amplitude were also qualified by a significant two-ways interaction between 'Shape' and 'Hemisphere' [F(2, 29) = 3.33, p < 0.05; ε = 0.79], independent of location-relevance. Further thorough analyses indicated that, overall, the shape-relevant condition attained a smaller mean amplitude response than the shape-irrelevant status in the left-, but not the right-hemisphere. This left-sided lateralization for the processing of the shape-relevant condition at this latency range can be appreciated both in the ERP waveforms drawn in Figure 3, and in the SCD mapping time series depicted in Figure 4A.

#### N1 (140-160 ms)

'Location' relevance affected N1, it being larger to image-pairs falling at the attended than unattended location [F(1, 17) = 5.67, p < 0.031; L+ = -1.73 μV, SD = 0.58 vs. L- = -0.99 μV; SD = 0.59] (see Figure 2 again). Furthermore, and most interestingly, the effects on N1 mean amplitude also indicated a significant triple interaction between 'Shape relevance', 'Hemisphere' and 'Electrode' [F(5, 81) = 2.32, p < 0.048; ε = 0.78]. This interaction was due to a larger mean amplitude for the shape-relevant condition than for the other two shape-relevance modes at the mesial- and lateral-occipital leads over the left-hemisphere only. This interaction also revealed that, independent of location relevance and electrode, over the right-hemisphere the N1 mean amplitude was, instead, more negative to both shape-relevant and shape-irrelevant pairs than distracters. Overall, these data patterns are numerically epitomized in Table 3.

**Table 3:** N1 mean amplitude values (μV) and Standard Deviations (in Italics) recorded at the posterior mesial- (mes-Occ) and lateral-occipital (lat-Occ) sites of the left and right hemispheres as a function of shape-relevance conditions.

	Hemisphere			
	
	Left Hemisphere		Right Hemisphere	
	**Electrode**		**Electrode**	
**Shape** **Relevance**	**mes-Occ**	**lat-Occ**	**mes-Occ**	**lat-Occ**
S+	-0.50	-0.32	-0.37	-0.18
	*0.50*	*0.62*	*0.70*	*0.65*
S+/-	-0.26	-0.12	-0.28	0.03
	*0.54*	*0.65*	*0.72*	*0.71*
S-	-0.26	-0.14	-0.40	-0.20
	*0.49*	*0.60*	*0.69*	*0.65*

S+ = Shape-relevant, S+/- = Shape relevant and irrelevant distracter, and S- = Shape-irrelevant

#### P300/N400 (380-450 ms)

**Figure 6:**
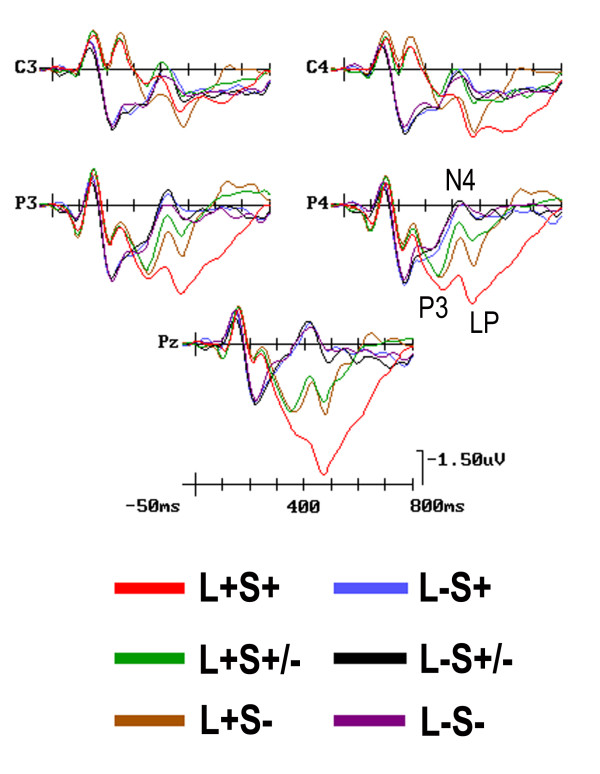
**Grand-average ERP waveforms recorded at left and right central and parietal scalp sites, as well as mesial-parietal sites, as a function of conjoined attentional conditions**. Unlike for previous figures, the waveforms have been plotted with a full time scale of 800 ms and tick-marks progressions of 100 ms to show the late latency attention effects. As can be seen, both P300 and N400 were strongly modulated by both shape- and location-relevance (*per se *and in interaction), whereas LP was overall more strongly modulated by image-pairs targetness.

At this late-latency stage, location relevant pairs elicited much larger P300 than irrelevant ones [F(1, 17) = 14.01, p < 0.001; L+ = 2.780 μV; L- = -0.256 μV), as unequivocally shown by the ERP waveforms of Figure 6. This component was also affected by shape relevance [ F(2, 34) = 8.972, p < 0.001; ε = 1] with large P300s to shape-relevant stimuli (S+ = 2.09 μV; SD = 0.59), intermediate for distracters (S+/- = 0.39 μV, SD = 0.68) and large N400s for attentionally inconspicuous pairs (S- = -1.03 μV, SD = 0.68). The interaction of 'Shape relevance × Electrode' [F(4, 68) = 4.01, p < 0.001; ε = 1] and the post-hoc comparisons showed that shape-relevance affected more robustly ERPs at parietal than anterior sites. The further interaction of 'Location × Shape relevance' [F(2, 34) = 5.94, p < 0.002; ε = 0.99] indicated that shape relevance was stronger at the relevant than the irrelevant location, with a much larger N400 to both shape-irrelevant pairs (S-) and distracters (S+/-) than targets (S+) at the spatially relevant location.

#### Late Positivity (LP, 450-520 ms)

This late-latency positive deflection was much greater to stimuli presented at the relevant (L+ = 4.5 μV, SD = 0.89) than the irrelevant (L- = 1.29 μV, SD = 0.60) spatial location (F(1, 17) = 25.31, p < 0.0000). Moreover, it was affected by 'shape relevance' (F(2, 34) = 8.68, p < 0.001; ε = 0.99), with larger LP to S+ (4.03 μV, SD = 0.72) than S- (2.99 μV, SD = 0.62) pairs, and intermediate amplitudes for distracters (S+/- = 2.37 μV, SD = 0.65). An interaction of 'Location × Shape relevance' (F(2, 34) = 10.02; p < 0.0004; ε = 1) pointed out shape relevance effects only at the attended location, where S+ elicited a LP of higher amplitude (6.81 μV, SD = 1.01) than both S- (4.78 μV, SD = 0.79; p < 0.03) and S+/- (3.15 μV, SD = 0.95; p < 0.0000), the latter not being, however, significantly different from S-.

## Discussion

In this experiment we found that, besides P1 response (80-120 ms) over the lateral-occipital areas, attention to both spatial and non-spatial features was able to modulate early sensory processing, as indexed by ERPs, at a latency and with a topography consistent with the earliest visual C1 response (60-100 ms) over mesial-occipital areas. This earliest mesial activity, relative to the first time window (60-80 ms), showed an increase in negativity for shape relevant stimulus pairs independent of the location relevance. It must be admitted that we cannot be absolutely certain that these earliest attention effects (60-80 ms) truly reflect the previously reported C1 having its origin in the occipital calcarine fissure and inverting in polarity with stimulation of the upper and lower occipital cortical banks of this fissure, since, rather than having followed this stimulation mode, our lateralized stimuli fell centered over the horizontal meridian of the visual field and extended within both the upper and lower quadrants of the left or right sides of the visual field. Despite this potential caveat, we believe that this earliest attentional modulation may actually reflect true C1 effects. Indeed, notwithstanding the uncrossed stimulation mode, there is a remarkable consistency between the precocious increase in negativity in the present study and the increase in negativity of the earliest C1 sensory response (N80, 40-80 ms) for attended than unattended spatial frequency gratings presented across the four quadrants of the visual field found by a recent report [32]. A LORETA source inverse solution performed on the difference wave obtained subtracting frequency irrelevant from frequency relevant stimuli also identified the active sources of the early attention effects in the visual primary cortex (BA17), the lateral occipital area (BA19), the superior parietal lobule (BA7), and various dorsolateral prefrontal regions. In the light of the consistency between the present earliest object-attention effects and the previous feature-related C1 attention effects in the crossed quadrants study, we believe that, rather than bring confounds to the debate on early attention modulation, the present results also add on to previous findings of C1 attention effects in the literature by providing evidence that, besides stimulus features, attention to more complex targets too, such as B/W drawings of familiar objects, can modulate the activation of the striate cortex at the earliest level, thus probably enhancing the processing of objects local details enabling the precocious selection.

The present data also showed an earliest effect of location relevance on sensors activity, in terms of an increased positivity (P80) to location relevant pairs, independent of their shape relevance or semantic category, starting as early as 60 ms post-stimulus, or earlier. It is interesting that despite the use of complex shapes, this finding is highly consistent with previous findings by both our own [29,30,32], and other research groups [23,28] using tasks requiring a voluntary allocation of spatial attention and simple stimulus features administered across the horizontal meridian. The early timing of this attentional modulation with reference to the post-stimulus onset is consistent with a source in the sensory occipital cortex, here unfortunately not directly corroborated by the use of any source reconstruction procedure. It is also worth noting that, despite the consistency of the present spatially-based C1 modulation with that found in a study by Fu et al. [20], at least in terms of early timing, the involuntary (or reflexive) nature of spatial attention allocation requested by the task used in that study opens some questions about this consistency, and the differential influences of voluntary and involuntary allocation of spatial attention on early visual sensory processing, which will have to be answered by means of further research.

It is interesting to note that shape and location relevance manifested in different polarities as the attentional modulation was concerned: paying attention to object features increased the negative voltage, whereas paying attention to spatial location increased the positive voltage of both C1 and P1 components. These findings support the notion that the space-based and the object-based attentional mechanisms are partially anatomically and functionally segregated [54,55]. In agreement with previous findings [34,39,30,56,30], the present data bring to light an intriguing dissociation: indeed, the ascending part of C1 component was more sensitive to shape relevance *per se *at the mesial occipital sites, closer to the visual striate area, while the descending portion of the same component was more sensitive to the combined interaction of the two features, as indicated by the interaction between shape and location relevance. And, indeed, it is possible that the initial portion of C1 component might have a stronger striate component, whereas the second, later-latency portion of C1 might reflect the later contribution of the extrastriate visual cortex, which is known to generate the P1 response, and be responsible of its space-based modulation [57,19]. Overall, these findings suggest that visual selective attention is able to modulate neural processing of object features independent of spatial processing, leading to the conclusion of a neuro-anatomical distinction of the 'what' and 'where' neural pathways [58]. A conclusion that is strongly supported also by empirical evidence of a significant interaction between voluntary visuospatial attention and perceptual load at target discrimination processing level, reflected at the scalp surface by N1 component, as a result of the activation of brain temporoparietal-occipital (TPO) junction [19].

An important theoretical issue to consider here is that our present findings clearly established that, as a result of the conjoined spatial and non-spatial attentional selection required by our task, visual cortex responsivity (including V1 activation) was cued to enhance the analysis of target shape attributes at both the relevant and irrelevant space locations, while simultaneously allocating spatial attention to the relevant target location, from the earliest post-stimulus processing time. This run counter to the traditional views of visual processing regarding spatial attention as having a special status, and spatial localization of visual input as preceding any feature- or object-selection carried out for the selective exploration of the outer world. At this regard, however, there has been an accumulation of empirical evidence from visual brain studies in the last two decades that supports the view that, on the one hand, the two selection mechanisms may operate in parallel right from the earliest levels of analysis, rather than being preceded by a space selection, and, on the other hand, feature-directed attention might directly precede attention to location, as repeatedly demonstrated in visual search studies. Indeed, there is a straightforward consistency between previous findings of our own research group in studies aimed at investigating neural mechanisms of spatial and non-spatial features (i. e., spatial frequency) conjoined selection [25,29,32], and the parallel processing view. Furthermore, evidence from single unit studies in monkeys demonstrated that object or feature-selection, rather than being preceded by a space selection, is centred "on line" on precise spatial coordinates allowing an "object-based space selection" [e. g., [59]]. Additional single unit evidence by Motter [60] and Treue and Martinez-Trujillo [61] have indicated that attention may be allocated to non spatial features in a location independent manner. This finding has been confirmed in humans by means of functional magnetic resonance (fMRI) imaging by Saenz et al [62]. As for the view of a possible precedence of feature-selection over the spatial selection, most fascinatingly combined event-related potentials (ERP) and event-related magnetic fields (ERFM) indexes of activations to task relevant features, starting about 140 ms after stimulus onset, independent of location relevance, have been reported in human volunteers performing a visual search task in which the spatial distribution of non-target items with relevant features was varied independently of the relevance of the location of the target. These activations were followed by a later lateralized response (the so called N2pc component) reflecting the deployment of attention to target location, which began at about 170 ms after stimulus input [63]. More recently, the measurement of N2pc during visual search tasks also revealed that there were functional differences in the deployment of attention to objects and space locations as a function of object-related structural conformation of space location, in that attention was shifted to a cued location in anticipation of a target shape when the location was marked by a placeholder object, whereas it was not when these cued locations were devoid of the placeholders, thus indicating a deployment of attention directly to objects [64].

It must be said that our C1 effects are somewhat earlier than both the feature-related response, starting at about 140 ms, and the N2pc effects (starting at 170 ms). However, it seems plausible that, inasmuch as the visual search entails a larger, time-consuming set of neural processes underlying stimulus features detection, preceding stimulus selection and recognition, than our own conjoined selection task, and, inasmuch as ERPs can properly index the timing of neural processing, our earlier-latency effects may possibly spring from the lack of any previous search of object features location to comply with the spatial and non-spatial features conjoined selection task.

A further point deserving discussion concerns our findings of independent effects for object-features and spatial location selection at the earliest C1 rising time range (i. e., 60-80 ms), and of interactive effects of these conjoined features, already starting in the C1 peak and/or descending time range (i. e., 80-100 ms) and increasing as visual input attentional processing progressed in time, as reflected at the scalp by the later-latency ERP components. Most fascinatingly, these findings seems to indicate that features-conjoined selection is obtained as a result of concurrent operations of multiple, narrowing levels of task-related attentional selectivity having distinctive properties and based on a progressively greater amount and a better quality of overall information about relevant input features, some of which at an higher order level than the C1 were here mirrored by the trend of the relatively-late latency and late-latency components.

At the N1 level, the attentional effects not only reflected location-relevance, as reported by previous literature [65,29], but also object-features effects. The latter effects showed to be characterized by complex attention-related hemispheric asymmetries. In fact, at the right-hemisphere, N1 was, overall, of greater amplitude for both the congruent shape-relevance (S+ and S-) conditions than for the mixed one (S+/-), thus plausibly reflecting the role of the right-sided ventral stream in the categorization of familiar shapes [66] independent of attention selection, consistently with the findings of a previous ERPs study [45] showing that the categorization effects between homomorphic, animal entities with faces and legs, and artefacts emerged at ~150 ms (N120-180 ms) over the right occipital-temporal sites. Conversely, at the left hemisphere an attentional selectivity between the relevant (S+) and irrelevant (S-) shapes was evident at the mesial- and lateral-occipital leads. Compatibly with the earlier left-sided P1 shape-selection effects observed, the present N1 hemispheric asymmetries in attentional selectivity seem to suggest that the left-hemisphere carries out a sharper sensory-perceptual selective processing across attentional relevance conditions than the right hemisphere. This mesh closely with the view of a predominance in attention selectivity of the left-hemisphere [e.g., [67]], in line with the accepted cognitive model of the latter hemisphere having a narrower attention focus and a more analytic attention strategy [68,69].

Most probably, the late positive (LP) component, reaching its maximum amplitude over the parietal sites, reflected the highest level of combined object and spatial processing, in terms of target selection and awareness, as well as decision making processes. This component is likely to reflect stimulus categorization processes and the attentive effects due to the interaction between shape and location relevance. The lack of any attentional modulation for shape-relevant stimuli at the irrelevant location seems to point out that outside the focus of spatial attention irrelevant stimuli are suppressed before being processed at the highest cognitive level [at this regard see [29]].

The finding that location relevance affected LP amplitude at parietal sites fully meshes with both the classic electrophysiological reports of a larger late positive complex (LPC) to attended than unattended spatial targets [e. g., [70,29]], and the parietal activations shown by blood-flow studies during the covert shifting of visuospatial attention [e. g., [71]]. However, activation of this same area has also been found in a feature conjunction search task [72], and in a divided attention task involving global and local processing [73], suggesting that this region is involved in more than shifting attention to a space location. In fact, it has also been demonstrated that the right superior medial parietal cortex is involved in overt and covert attention tasks of object- and space-based interactions [74]. In agreement with these findings, our results of the N400 and LP components indicate an interaction of space and object feature processing over parietal and central cortex. In particular, N400 component may be possibly conceived as a mismatch response sensitive to the processing of semantically incongruent stimuli [75].

There are other important theoretical issues to be considered here. Although the differences found across the various shape-relevance conditions at C1 level in no way index object categorization processes *per se*, these conditions being simply a reflection of the different degree of their attentional saliency, it has to be considered here that, besides in attentional-relevance, the distracter pairs (S+/-) also differed somewhat in terms of stimulus-category features from those of both the salient (S+) and inconspicuous (S-) attention conditions. In our view, then, it is reasonable to believe that the significant differences in neural processing levels found within the relevant location between the salient condition (L+S+) and the distracting one (L+S+/-), besides the inconspicuous one (L+S-), might indicate that the visual system is somehow able to distinguish, at a first basic, unconscious level, between images of different semantic categories already at the earliest sensory processing level. Indeed, objective evidence in the literature seems to support this claim. On the one hand, man-made categories have more energy in 'cardinal' (i.e., vertical and horizontal) orientations compared to natural categories [76]. On the other hand, animals have been indicated to be more 'homomorphic' (i.e., they all have heads and eyes that are generally round, and legs) than artefacts, that tend to contain more rectilinear strokes [77]. Additionally, and most importantly, faces and man-made objects naturally vary in their Fourier spatial frequency amplitude spectrum (AS), with a steeper spectrum decrease for faces compared to natural images [78]. Consistent with the latter evidence, proofs have also been provided that rapid image recognition can be biased by simply priming the amplitude spectrum information [79,80]. All in all, these indications strongly support the view that the human brain may be intrinsically tuned to this low-level information characterizing faces and body parts, thus facilitating rapid 'homomorphic' traits detection.

In line with all these indications, it is not unlikely that, despite the compensation for average luminance, size and other visual features, our animal and artefact categories too, besides the distracter pairs, still differed from each other in all the aforementioned basic features, but, in all prospect, mostly in their spatial frequency amplitude spectrum. It is possible, then, that these basic informational differences between natural and man-made objects *per se*, may account for our early C1 effects for animals Vs artefacts discrimination. At these regards, far from being surprising, the attention effect found as earliest as 60 millisecond in C1 is, in our view, absolutely consistent with the findings of earliest attentional effects by both our own [29,30,32] and other groups' previous studies involving single features, such as spatial frequencies, spatial attention, emotional faces, etc., differing between a target and distracters.

Most importantly, strong support to the aforementioned claims derives from most recent ERPs experimental evidence. Indeed, to investigate how fast the human brain categorizes faces in comparisons to other visual stimuli, Rossion and Caharel [81] asked a sample of volunteers to discriminate between pictures of faces and cars, presented in both their intact and phase-scrambled versions, counterbalanced for luminance and other visual features. The authors found discriminative effects - a larger response to pictures of faces than cars independent of shape versions - at latency stages earlier than 100 ms (80-100 ms), indicated by them, in their own terms, as "a very early P1 level". However, a later N170 component also showed to be larger for faces than cars, but for the intact shape versions only. Overall, they explained their early-latency P1 effects to faces as a brain response to low-level visual cues, namely the steeper Fourier amplitude spectrum (AS) of face images indicated by Keil [78], and their N170 effects as a scalp-recorded reflection of a true face perception or categorization stage.

That the visual system might give signs of distinguishing between stimulus categories at the earliest 60-80 processing latency range, as we found, does not absolutely mean that it has reached the complete recognition of the different stimulus categories at conscious level at this processing time. Quite on the contrary, as supported by Rossion and Caharel [81], besides our own findings, it may simply indicate that the system has begun the selection of salient perceptual information at an 'entry' level required, as a prerequisite, for detecting, identifying, and categorizing objects by means of different perceptual decisions, the latter being very probably based on different, successive levels of accumulation of salient information and different time scales. This would be consistent with both the views that basic-level categorization is an entry level of processing that precedes stages of categorization at other levels [82] very likely carried out through feedforward processing [48], and that conscious perception is possible only with recurrent processing of the stimulus input as advanced by Roelfsema and colleagues [83,84]. Indeed, counter to traditional views of object visual processing evidence has stemmed that our visual system is able to categorize images of natural visual scenes at remarkable speed [85]. At this regard, Kirchner and Thorpe [86] have most impressively demonstrated that the participants of their study were able to perform a speeded saccade toward one out of two pictures presented, which contained an animal target, as fast as 120 ms post-stimulation. On the other hand, counter to traditional views of object detection and categorization, a parallel line of research over the past few years has also shown that objects can be often successfully detected without being successfully categorized [e. g., [87,88]]. Besides, other studies have shown that stimulus material manipulations, as stimulus inversion and image degradation, impair categorization at a more basic-level but not object detection [e. g., [89]]. In addition, and most fascinatingly, several recent studies have also demonstrated effects of feature-based attention on the processing of stimuli of which the participants where not at all aware [e. g., [90,91]], in line with the view proposed by different sources that different neurophysiological processes are underlying attention and awareness [e. g., [92,93]].

It is worth noting that both our present earliest C1 and P1 shape-relevance effects, and the pre-100 ms P1 face effects observed in ERPs by Rossion and Caharel [81], which are in all likelihood directly related to the low-level visual cues of the stimuli [76-78], and most probably their Fourier AS information [78], meshes closely not only with the timing of the fastest saccadic behavioural response, but also with the differences observed between the detection and categorization processes, as well as between the neurophysiological processes underlying attention and awareness. For truth's sake, however, it must be reminded that, notwithstanding these intriguing compatibilities, the whole of previous ERP studies indexing the timing of shapes categorization has indicated category-divergence effects at the relatively later level of N1 (i. e., 140-160 ms) component [43-45].

A further source of evidence supporting our present claims concerns our behavioural findings. Indeed, no matter their category difference, our participants' motor response time to both animal and artefact categories occurred not only at a much later time than C1 latency range, but also somewhat later than both P300/N400 and LP processing levels too. Moreover, participants' response errors (FAs) almost exclusively concerned distracter pairs at the relevant spatial location (L+S+/-). Overall, this seem to further support the viewpoint that the significant effects at the C1 processing level might reflect a first basic-level selection of object salient features and spatial localization used to drive a perceptual decision process to which the relative later timing of motor responses, indexing target true conscious recognition and categorization, would be related. These later processes would be based on the availability of a greater amount and a better quality of perceptual evidence [see [87] for a review advancing such theoretical hypothesis]. Despite the consistency with all this evidence from different lines of research on visual attentional and perceptual processing, we want anyway to point out that these claims must be confirmed by further research.

A most important matter also deserves to be discussed. Indeed, there seems to be a close consistency between the present C1 shape effects and the C1 feature effects of a previous study of our group, which, using different spatial-frequency gratings presented in the four quadrants of the visual space during a spatial and featural conjoined-selection task [32], showed that these C1 feature effects had a source in the primary, besides the secondary, visual areas. In the light of the aforementioned consistency, we are akin to advance the intriguing hypothesis that there may be a similar involvement of the primary visual cortex in the shape-selection-related C1 effects obtained in the present study. Our hypothesis seems to borrow strong support from some recent influential evidence in the literature. Notably, single cells recordings in macaque V1 have undoubtedly demonstrated that the neuronal micro-networks of this low-level occipital area not only may actively merge the line and edge components of the visual scenes into perceptually unified wholes [e. g., [94]], but are also involved in the top-down gating of horizontal connections of this area through feedback projections inhibiting some sets of lateral interactions and/or activating others during geometric selectivity, rather than a simple gain control or a simple reflection of higher sensory processing [e. g., [95]]. Most interestingly, these distinct selectivity patterns between the task conditions would begin to develop between 70 ms and 120 ms after stimulus onset, and would reach maturity between 110 and 160 ms, the latter latency ranges being pretty consistent with our ERPs earliest- and early latency effects.

In summary, our data provide evidence of an early modulation of brain activity (~ 60 ms) over the mesial and lateral occipital cortices for both location and shape attentional relevance. While, on the one hand, target processing increased brain bioelectrical activity, which resulted as an increased N80 response for shape relevant pairs at mesial occipital sites (striate cortex), and as an increased P80 for location relevant stimuli at lateral occipital sites, on the other hand, non-targets (both distracters, but especially irrelevant pairs) elicited a decreased neural response, and stimuli falling outside the attentive focus were ignored at the highest cognitive levels immediately preceding the motor response.

All in all, this is one of the first pieces of experimental evidence in humans indicating that, besides other brain occipital areas, V1 area may also be directly involved in object selection. This involvement would start since the earliest post-stimulus processing latency, by contributing with the analysis of basic information (possibly curved vs. straight lines, presence of little circles, etc.), and most of all, possibly, of the Fourier spatial frequency amplitude spectrum, thus suggesting that visual attention can start modulating visual processing to a much earlier stage than previously thought [4].

## Limitations

The potential limitations of this study are; (1) our stimulus materials were not presented across the horizontal meridian of the visual field so to have an inversion in polarity of the earliest C1 sensory response, renown in the literature to reflect at the scalp surface the activation of the visual striate areas; and (2) due to the lack of any source reconstruction, the activity could not be precisely localized in the striate cortex. Thus, our conclusions are based on the findings of robust differences in amplitude between relevant and irrelevant shapes, besides potential conflicting distracters, at the earliest time course of post-stimulus neural processing, namely in the latency range of the C1 component.

## List of abbreviations

EEG: Electroencephalogram; ERMF: Event-related magnetic fields; ERP: Event-related potentials; FAs: False alarms; LP: Late positivity; SCD: Scalp current density

## Authors' contributions

AZ and AMP took part in designing and planning the experiment, data analyses and manuscript preparation. Both authors also contributed to figure preparation and computational analyses for topographical mapping. Again, both of them read and approved the final manuscript.

## References

[B1] Martínez A Anllo-Vento L Sereno MI Frank LR Buxton RB Dubowitz DJ Wong EC Hinrichs H Heinze HJ Sa H Involvement of striate and extrastriate visual cortical areas in spatial attention Nature Neurosci 1999 2 364 369 10.1038/7274 10204544

[B2] Martínez A Di Russo F Anllo-Vento L Sereno MI Buxton RB Hillyard SA H Putting spatial attention on the map: timing and localization of stimulus selection processes in striate and extrastriate visual areas Vision Res 2001 41 1537 1547 10.1016/s0042-6989(00)00267-4 11322985

[B3] Baas JM Kenemans JL Selective attention to spatial frequency: an ERP and source localization analysis Clin Neurophysiol 2002 13 1840 1854 12417240 10.1016/s1388-2457(02)00269-9

[B4] Hillyard SA Anllo-Vento L Event-related brain potentials in the study of visual selective attention Proc Natl Acad Sci USA 1998 95 781 10.1073/pnas.95.3.781 9448241 PMC33798

[B5] Grunewald A Bradley DC Andersen RA Neural Correlates of Structure form-Motion Perception in Macaque V1 and MT J Neurosci 2002 22 6195 6207 12122078 10.1523/JNEUROSCI.22-14-06195.2002 PMC6757912

[B6] Luck SJ Chelazzi L Hillyard SA Desimone R Neural Mechanisms of Spatial Selective Attention in Areas V1, V2, and V4 of Macaque Visual Cortex J Neurophysiol 1997 77 24 42 9120566 10.1152/jn.1997.77.1.24

[B7] Aine CJ Supek S George JS Temporal dynamics of visual-evoked neuromagnetic sources: effects of stimulus parameters and selective attention The Internat J Neurosci 1995 80 79 104 10.3109/00207459508986095 7775063

[B8] Treue S Neural correlates of attention in primate visual cortex Trends Neurosci 2001 24 295 300 10.1016/S0166-2236(00)01814-2 11311383

[B9] Ito M Gilbert CD Attention modulates contextual influences in the primary visual cortex of alert monkey Neuron 1999 22 593 604 10.1016/S0896-6273(00)80713-8 10197538

[B10] Motter BC Focal attention produces spatially selective processing in visual cortical areas V1, V2, and V4 in the presence of competing stimuli J Neurophysiol 1993 70 909 919 8229178 10.1152/jn.1993.70.3.909

[B11] McAdams CJ Reid RC Attention modulates the responses of simple cells in monkey primary visual cortex J Neurosci 2005 25 11023 11033 10.1523/JNEUROSCI.2904-05.2005 16306415 PMC6725881

[B12] McAlonan K Cavanaugh J Wurtz RH Guarding the gateway to cortex with attention in visual thalamus Nature 2008 456 391 395 10.1038/nature07382 18849967 PMC2713033

[B13] Gandhi SP Heeger DJ Boynton GM Spatial attention affects brain activity in human primary visual cortex Proc Natl Acad Sci USA 1999 96 3314 3319 10.1073/pnas.96.6.3314 10077681 PMC15939

[B14] Kastner S Pinsk MA De Weerd P Desimone R Ungerleider LG Increased Activity in Human Visual Cortex during Directed Attention in the Absence of Visual Stimulation Neuron 1999 22 751 761 10.1016/S0896-6273(00)80734-5 10230795

[B15] Silver MA Ress D Heeger DJ Neural Correlates of Sustained Spatial Attention in Human Early Visual Cortex J Neurophysiol 2007 97 229 237 10.1152/jn.00677.2006 16971677 PMC1868502

[B16] Shulman GL Corbetta MJA Fiez JA Buckner RL Miezin FM Raichle ME Petersen SE Searching for activations that generalize over tasks Hum Brain Mapp 1997 5 317 322 10.1002/(SICI)1097-0193(1997)5:4<317::AID-HBM19>3.0.CO;2-A 20408235

[B17] Slotnick SD Schwarzbach J Yantis S Attentional inhibition of visual processing in human striate and extrastriate cortex Neuroimage 2003 19 1602 1611 10.1016/S1053-8119(03)00187-3 12948715

[B18] Zani A Proverbio AM L Itti G, Rees JK, Tsotsos The timing of attentional modulation of visual processing as indexed by ERPs Neurobiology of attention 2005 San Diego (USA): Academic Press 514 519

[B19] Fu S Zinni M Squire PN Kumar R Caggiano DM Parasuraman R When and where perceptual load interacts with voluntary visuospatial attention: An event-related potential and dipole modeling study Neuroimage 2008 39 1345 1355 10.1016/j.neuroimage.2007.09.068 18006335

[B20] Fu S Huang Y Wang Y Fedota J Greenwood PM Parasuraman R Perceptual load interacts with involuntary attention at early processing stages: event-related potential studies Neuroimage 2009 48 191 199 10.1016/j.neuroimage.2009.06.028 19539769 PMC2861284

[B21] Fu S Fedota JR Greenwood PM Parasuraman R Dissociation of visual C1 and P1 components as a function of attentional load: An event-related potential study Biol Psychol 2010 85 171 178 10.1016/j.biopsycho.2010.06.008 20599467 PMC2921581

[B22] Kelly SP Gomez-Ramirez M Foxe JJ Spatial Attention Modulates Initial Afferent Activity in Human Primary Visual Cortex Cereb Cortex 2008 18 2629 2636 10.1093/cercor/bhn022 18321874 PMC2733320

[B23] Rauss KS Pourtois G Vuilleumier P Schwartz S Attentional load modifies early activity in human primary visual cortex Human Brain Mapping 2009 30 1723 1733 10.1002/hbm.20636 18711710 PMC6871007

[B24] Zani A Proverbio AM ERP signs of early selective attention effects to check size Electroencephalogr Clin Neurophysiol 1995 95 277 92 10.1016/0013-4694(95)00078-D 8529559

[B25] Zani A Proverbio AM Attention modulation of short latency ERPs by selective attention to conjunction of spatial frequency and location J Psychophysiol 1997 11 21 32

[B26] Proverbio AM Esposito P Zani A Early involvement of temporal area in attentional selection of grating orientation: an ERP study Cogn Brain Res 2002 13 139 151 10.1016/S0926-6410(01)00103-3 11867258

[B27] Khoe W Mitchell JF Reynolds JH Hillyard SA Exogenous attentional selection of transparent superimposed surfaces modulates early event related potentials Vision Res 2005 45 3004 3014 10.1016/j.visres.2005.04.021 16153678

[B28] Karns CM Knight RT Intermodal auditory, visual, and tactile attention modulates early stages of neural processing J Cogn Neurosci 2008 21 669 683 18564047 10.1162/jocn.2009.21037 PMC3092632

[B29] Zani A Proverbio AM J Dupri ERP signs of frontal and occipital processing of visual targets and distracters within and without the channel of spatial attention Focus on Neuropsychology Research 2006 New York: Nova Science Publishers 38 88

[B30] Zani A Proverbio AM Selective attention to spatial frequency gratings affects visual processing as early as 60 msec poststimulus Percept Mot Skills 2009 109 140 158 10.2466/pms.109.1.140-158 19831095

[B31] Zani A Proverbio AM Attention modulation of C1 and P1 components of visual evoked potentials [abstract] EEG Clin Neurophysiol 1997 103 97

[B32] Proverbio AM Del Zotto M Zani A Electrical neuroimaging evidence that spatial frequency-based selective attention affects V1 activity as early as 40-60 ms in humans BMC Neurosci 2010 11 59 10.1186/1471-2202-11-59 20459601 PMC2890012

[B33] Luck SJ Hillyard SA Mouloua M Woldorff MG Clark VP Hawkins HL Effects of spatial cuing on luminance detectability: Psychophysical and electrophysiological evidence for early selection J Exp Psych: Hum Perc Perform 1994 20 887 904 10.1037//0096-1523.20.4.887 8083642

[B34] Proverbio AM Del Zotto M Zani A Inter-individual differences in the polarity of early visual responses and attention effects Neurosci Let 2007 419 131 136 10.1016/j.neulet.2007.04.048 17490815

[B35] Stolarova M Keil A Moratti S Modulation of the C1 Visual Event-related Component by Conditioned Stimuli: Evidence for Sensory Plasticity in Early Affective Perception Cer Cortex 2006 16 876 887 10.1093/cercor/bhj031 16151178

[B36] Pourtois G Grandjean D Sander D Vuilleumier P Electrophysiological Correlates of Rapid Spatial Orienting Towards Fearful Faces Cereb Cortex 2004 14 619 633 10.1093/cercor/bhh023 15054077

[B37] Proverbio AM Riva F Zani A Martin E Is it a baby? Perceived Age Affects Brain Processing of Faces Differently in Women and Men J Cog Neurosci 2011 23 3197 3208 10.1162/jocn_a_00041 21557651

[B38] Proverbio AM Adorni R Zani A Trestianu L Sex differences in the brain response to affective scenes with or without humans Neuropsychol 2009 47 2374 2388 10.1016/j.neuropsychologia.2008.10.030 19061906

[B39] Proverbio AM Adorni R C1 and P1 visual responses to words are enhanced by attention to orthographic vs lexical properties Neurosci Let 2009 463 228 233 10.1016/j.neulet.2009.08.001 19664687

[B40] Rauss K Schwartz S Pourtois G Top-down effects on early visual processing in humans: A predictive coding framework Neurosci Biobehav Rev 2011 35 1237 10.1016/j.neubiorev.2010.12.011 21185860

[B41] Scerif G Worden MS Davidson M Seiger L Casey BJ Context Modulates Early Stimulus Processing when Resolving Stimulus-Response Conflict J Cog Neurosci 2006 18 781 792 10.1162/jocn.2006.18.5.781 16768377

[B42] Taylor MJ Non-spatial attentional effects on P1 Clin Neurophysiol 2002 113 1903 1908 10.1016/S1388-2457(02)00309-7 12464327

[B43] Thorpe S Fize D Marlot M Speed of processing in the human visual processing Nature 1996 381 520 522 10.1038/381520a0 8632824

[B44] Tanaka J Luu P Weisbrod M Kiefer M Tracking the time course of object categorization using event-related potentials NeuroRep 1999 10 829 835 10.1097/00001756-199903170-00030 10208556

[B45] Proverbio AM Del Zotto M Zani A The emergence of semantic categorization in early visual processing: ERP indices of animal vs artifact recognition BMC Neurosci 2007 8 24 doi: 10.1186/1471-2202-8-24 10.1186/1471-2202-8-24 17411424 PMC1852317

[B46] Wijers AA Mulder G Okita T Mulder LJM An ERP study on memory search and selective attention to letter size and conjunctions of letter size and color Psychophysiol 1989 26 529 547 10.1111/j.1469-8986.1989.tb00706.x 2616701

[B47] Mangun GR Hillyard SA Luck SJ E Meyer, S Kornblum Electrocortical substrates of visual selective attention Attention and Performance XIV 1993 Cambridge, MA: The MIT Press 219 243

[B48] Schmidt T Haberkamp A Veltkamp GM Weber A Seydell-Greenwald A Schmidt F Visual processing in rapid chase systems: image processing, attention, and awareness Front Psychol 2011 2 doi: 10.33839/fpsyg.2011.00169 10.3389/fpsyg.2011.00169 PMC3139957 21811484

[B49] Yamagishi N Callan DE Goda N Anderson SJ Yoshida Y Kawato M Attentional modulation of oscillatory activity in human visual cortex Neuroimage 2003 20 98 113 10.1016/S1053-8119(03)00341-0 14527573

[B50] Rihs TA Michel CM Thut G A bias for posterior alpha-band power suppression versus enhancement during shifting versus maintenance of spatial attention Neuroimage 2009 44 190 199 10.1016/j.neuroimage.2008.08.022 18793732

[B51] Romei V Gross J Thut G On the role of prestimulus alpha rhythms over occipito-parietal areas in visual input regulation: correlation or causation? J Neurosci 2010 30 8692 8697 10.1523/JNEUROSCI.0160-10.2010 20573914 PMC6634639

[B52] Capotosto P Babiloni C Romani GL Corbetta M Frontoparietal cortex controls spatial attention through modulation of anticipatory alpha rhythms J Neurosci 2009 6 5683 5683 10.1523/JNEUROSCI.0539-09.2009 PMC2692025 19420253

[B53] Banerjee S Snyder AC Molholm S Foxe JJ Oscillatory alpha-band mechanisms and the deployment of spatial attention to anticipated auditory and visual target locations: Supramodal or sensory-specific control mechanisms? J Neurosci 2011 31 9923 9932 10.1523/JNEUROSCI.4660-10.2011 21734284 PMC3343376

[B54] Roelfsema PR Lamme VAF Spekreijse H Object-based attention in the primary visual cortex of the macaque monkey Nature 1998 395 376 381 10.1038/26475 9759726

[B55] Vecera SP Farah MJ Does visual attention select objects or locations? J Exp Psychol: Gen 1994 123 146 160 8014610 10.1037//0096-3445.123.2.146

[B56] Simon-Thomas ER Brodsky K Willing C Sinha R Knight RT Distributed neural activity during object, spatial and integrated processing in humans Cog Brain Res 2003 16 457 467 10.1016/S0926-6410(03)00060-0 12706225

[B57] Foxe JJ Simpson GV Flow of activation from V1 to frontal cortex in humans: a framework for defining "early" visual processing Exp Brain Res 2002 142 139 150 10.1007/s00221-001-0906-7 11797091

[B58] Wang J Zhou T Qiu M Du A Cai K Wang Z Zhou C Meng M Zhuo Y Fan S Chen L Relationship between ventral stream for object vision and dorsal stream for spatial vision: An fMRI+ERP study Hum Brain Map 1999 8 170 181 10.1002/(SICI)1097-0193(1999)8:4<170::AID-HBM2>3.0.CO;2-W PMC6873314 10619412

[B59] Olson CR Gettner S Brain representation of object-centered space Curr Opin Neurobiol 1996 6 165 170 10.1016/S0959-4388(96)80069-9 8725957

[B60] Motter BC Neural correlates of attentive selection for color or luminance in extrastriate area V4 J Neurosci 1994 14 2178 2189 8158264 10.1523/JNEUROSCI.14-04-02178.1994 PMC6577115

[B61] Treue S Martinez Trujillo JC Feature-based attention influences motion processing gain in macaque visual cortex Nature 1999 399 575 579 10.1038/21176 10376597

[B62] Saenz M Buracas GT Boynton GM Global effects of feature-based attention in human visual cortex Nat Neurosci 2002 5 631 632 10.1038/nn876 12068304

[B63] Hopf J-M Boelmans K Schenfeld MA Luck S Heinze H-J Attention to features precedes attention to locations in visual search: Evidence from electromagnetic brain responses in humans J Neurosci 2004 24 1822 1832 10.1523/JNEUROSCI.3564-03.2004 14985422 PMC6730400

[B64] Woodman GF Arita JT Luck S A cuing study of the N2pc component: An index of attentional deployment to objects rather than spatial locations Brain Res 2009 1297 101 111 19682440 10.1016/j.brainres.2009.08.011 PMC2758329

[B65] Clark VP Hillyard SA Spatial selective attention affects early extrastriate but not striate components of the visual evoked potentials J Cogn Neurosci 1996 8 387 402 10.1162/jocn.1996.8.5.387 23961943

[B66] Zhang Y Meyers EM Bichot NP Serre T Poggio TA Desimone R Object decoding with attention in inferior temporal cortex Proc Nat Acad Sciences 2011 108 8850 8855 10.1073/pnas.1100999108 PMC3102370 21555594

[B67] Reuter-Lorentz PA Kinsbourne M Moscovitch M Hemispheric control of spatial attention Brain Cogn 1990 12 240 266 10.1016/0278-2626(90)90018-J 2340154

[B68] Robertson LC Lamb MR Knight RT Effects of lesions of temporal-parietal junction on perceptual and attentional processing in humans J Neurosci 1988 10 3757 3789 3193178 10.1523/JNEUROSCI.08-10-03757.1988 PMC6569588

[B69] Palmer T Tzeng OJ Cerebral asymmetry in visual attention Brain Cogn 1990 13 46 58 10.1016/0278-2626(90)90039-Q 2346639

[B70] Mangun GR Hinrichs H Scholz M Mueller-Gartner HW Herzog H Krause BJ Tellman L Kemma L Heinze HJ Integrating electrophysiology and neuroimaging of spatial selective attention to simple isolated visual stimuli Vision Res 2001 41 1423 1435 10.1016/S0042-6989(01)00046-3 11322984

[B71] Corbetta M Miezin FM Shulman GL Petersen SE A PET study of visuospatial attention J Neurosci 1993 13 1202 1226 8441008 10.1523/JNEUROSCI.13-03-01202.1993 PMC6576604

[B72] Corbetta M Shulman GL Miezin FM Petersen SE Superior parietal cortex activation during spatial attention shifts and visual feature conjunction Science 1995 270 802 805 10.1126/science.270.5237.802 7481770

[B73] Fink GR Halligan PW Marshall JC Frith CD Frackowiak RS Dolan RJ Neural mechanisms involved in the processing of global and local aspects of hierarchically organized visual stimuli Brain 1997 120 1779 1791 10.1093/brain/120.10.1779 9365370

[B74] Fink GR Dolan RJ Halligan PW Marshall JC Frith CD Space-based and object-based visual attention: shared and specific neural domains Brain 1997 120 2013 2028 10.1093/brain/120.11.2013 9397018

[B75] Federmeier KD Kutas M Picture the difference: electrophysiological investigations of picture processing in the two cerebral hemispheres Neuropsychol 2002 40 730 747 10.1016/S0028-3932(01)00193-2 11900725

[B76] Torralba A Oliva A Statistics of natural image categories Network 2003 14 391 412 10.1088/0954-898X/14/3/302 12938764

[B77] Tranel D Damasio H Damasio AR A neural basis for the retrieval of conceptual knowledge Neuropsychol 1997 35 1319 1327 10.1016/S0028-3932(97)00085-7 9347478

[B78] Keil MS Does face image statistics predict a preferred spatial frequency for human face processing? Proc Royal Soci B: Biol Sci 2008 275 2095 2100 10.1098/rspb.2008.0486 PMC2603213 18544506

[B79] Guyader N Chauvin A Peyrin C Herault J Marendaz C Image phase or amplitude? Rapid scene categorization is an amplitude-based process CR Biologies 2004 327 313 318 10.1016/j.crvi.2004.02.006 15212363

[B80] Kaping D Tzvetanov T Treue T Adaptation to statistical properties of visual scenes biases rapid categorization Vis Cogni 2007 15 12 19 10.1080/13506280600856660

[B81] Rossion B Caharel S ERP evidence for the speed of face categorization in the human brain: Disentangling the contribution of low-level visual cues from face perception Vis Res 2011 51 12 1297 1311 10.1016/j.visres.2011.04.003 21549144

[B82] Grill-Spector K Kanwisher N Visual recognition: as soon as you know it is there, you know what it is Psychol Sci 2005 16 152 160 10.1111/j.0956-7976.2005.00796.x 15686582

[B83] Roelfsema PR Lamme VAF Spekreijse H Bosch H Figure-ground segregation in a recurrent network architecture J Cogn Neurosci 2002 14 525 537 10.1162/08989290260045756 12126495

[B84] Roelfsema PR Cortical algorithms for perceptual grouping Annu Rev Neurosci 2006 29 203 227 10.1146/annurev.neuro.29.051605.112939 16776584

[B85] Hedgè J Time course of visual perception: coarse-to-fine processing and beyond Prog Neurobiol 2008 84 405 439 10.1016/j.pneurobio.2007.09.001 17976895

[B86] Kirchner H Thorpe SJ Ultra-rapid object detection with saccadic eye movements: visual processing speed revisited Vision Res 2006 46 1762 1776 10.1016/j.visres.2005.10.002 16289663

[B87] Mack ML Palmeri TJ The timing of visual object categorization Front Psychol 2011 2 doi: 10.33839/fpsyg.2011.00165 10.3389/fpsyg.2011.00165 PMC3139955 21811480

[B88] de la Rosa S Chouderry RN Chatziastros A Visual object detection, categorization, and identification tasks are associated with different time courses and sensitivities J Exp Psychol Hum Percept Perform 2011 37 38 47 21038993 10.1037/a0020553

[B89] Mack ML Gauthier I Sadr J Palmeri TJ Object detection and basic level categorization: sometimes you know it is there before you know what it is Psychon Bull Rev 2008 15 28 35 10.3758/PBR.15.1.28 18605476

[B90] Shin K Stolte M Chong SC The effect of spatial attention on invisible stimuli Attn Percept Psychophys 2009 71 1507 1513 10.3758/APP.71.7.1507 19801611

[B91] Schmidt F Schmidt T Feature-based attention to unconscious shapes and colors Attn Percept Psychophys 2010 72 112 118 10.3758/APP.72.6.1480 20675795

[B92] Lamme VAF L Itti G, Rees JK Tsotsos The difference between visual attention and awareness: a cognitive neuroscience perspective Neurobiology of Attention 2005 San Diego: Elsevier Academic Press 167 174

[B93] Koch C Tsuchiya N Attention and consciousness: two distinct brain processes Trends Cogn Sci 2007 11 16 22 10.1016/j.tics.2006.10.012 17129748

[B94] Li W Piech V Gilbert CD Contour saliency in primary visual cortex Neuron 2006 50 951 962 10.1016/j.neuron.2006.04.035 16772175

[B95] McManus JNJ Li W Gilbert CD Adaptive shape processing in primary visual cortex Proc Natl Acad Sci USA 2011 108 9739 9746 10.1073/pnas.1105855108 21571645 PMC3116391

